# Rare Ocular Foe: A Case of Fungal Keratitis Caused by *Podospora bulbillosa*

**DOI:** 10.22034/cmm.2026.345427.1677

**Published:** 2026-07-29

**Authors:** Madhu Rai, Vaibhav Nagpal, Chaitali Nikam, Anirvan Chatterjee, Amrutraj Zade, Sourav Das, Savitri Sharma

**Affiliations:** 1 Ocular Microbiology Service, Shantilal Shanghvi Eye Institute, Wadala East, Mumbai, Maharashtra, India; 2 Junior Faculty, Cornea Service, Shantilal Shanghvi Eye Institute, Wadala East, Mumbai, Maharashtra, India; 3 Haystack Analytics Private Limited SINE, IIT Bombay, Powai, Mumbai, India; 4 Jhaveri Microbiology Centre, L V Prasad Eye Institute, Hyderabad, Telangana, India

**Keywords:** Dematiaceous fungus, Oxford nanopore sequencing, *Podospora bulbillosa*

## Abstract

**Background and Purpose:**

Fungal keratitis is commonly caused by filamentous fungi following trauma or environmental exposure. This study aimed to report a rare case of fungal keratitis confirmed using Oxford Nanopore Sequencing.

**Case presentation:**

A 25-year-old male developed pain and redness in his right eye after dust exposure. Slit-lamp examination showed a superficial corneal infiltrate with vision 20/50 in the affected eye. Microscopy revealed septate fungal filaments, and SDA culture produced rapidly growing colonies that turned black. Sequencing and phylogenetic analysis identified the fungus as *Podospora bulbillosa*. The patient responded well to 5% natamycin, achieving complete healing.

**Conclusion:**

The case reported here emphasized the susceptibility of the cornea to rare fungal pathogens and the importance of timely selection of appropriate antifungal therapy.

## Introduction

Fungal keratitis remains a major cause of corneal blindness worldwide and continues to be a significant public health concern due to diagnostic challenges and poor clinical outcomes [1]. In the past 20 years, dematiaceous fungi have become the third most common cause of fungal keratitis, after *Fusarium* and *Aspergillus species* [2-4]. With advances in molecular diagnostic techniques, these melanized molds are increasingly being reported as ocular pathogens [5]. Identification at the species level is essential to understand their diverse clinical presentations, disease progression, and therapeutic response. Species-specific treatment outcomes offer useful direction for selecting appropriate antifungal therapy [6].

In this report, a rare dematiaceous fungus, *Podospora bulbillosa*, was isolated from a corneal scraping of a patient diagnosed with fungal keratitis. Prompt laboratory identification enabled the early initiation of treatment, leading to rapid clinical improvement and complete recovery without surgical intervention. This case demonstrated the need for clinical suspicion toward dematiaceous fungi, the importance of timely microbiological diagnosis, and the value of early management in the achievement of favorable outcomes.

## Case presentation

A 25-year-old male residing at Wadala, Mumbai, presented to the cornea clinic with complaints of redness and pain in the right eye for the past 4–5 days. Visual acuity in the right eye was 20/50, while the left eye was 20/20. The patient reported an incident of an unknown foreign body falling into his right eye, followed by vigorous rubbing. There was no history of relevant systemic conditions.

Slit-lamp examination of the right eye revealed conjunctival congestion and a superficial anterior stromal corneal infiltrate measuring 5 mm x 3 mm with an epithelial defect measuring 2 mm x 2 mm (Figure 1a). The anterior chamber showed inflammation with 2+ cells, the fundus examination was normal, and the left eye was also normal. Under topical anesthesia, multiple corneal scrapings were collected from the right eye in the clinic under slit lamp magnification for microbiological processing. Direct microscopic examinations were performed by staining the smears with calcofluor white [10% KOH + 0.1% CFW] and Gram stain. Corneal scrapings were inoculated on 5% sheep blood agar, Sabouraud dextrose agar (SDA), and potato dextrose agar (PDA). The SDA and PDA plates were incubated at 25 °C, while the blood agar was incubated at 37 °C. The presence of septate, branched fungal filaments was observed in both the KOH+CFW mount and the Gram-stained smear (Figure 1b and c). Based on the findings, a provisional diagnosis of fungal keratitis was made, and treatment with 5% topical natamycin eye drops was initiated at hourly intervals.

**Figure 1:**
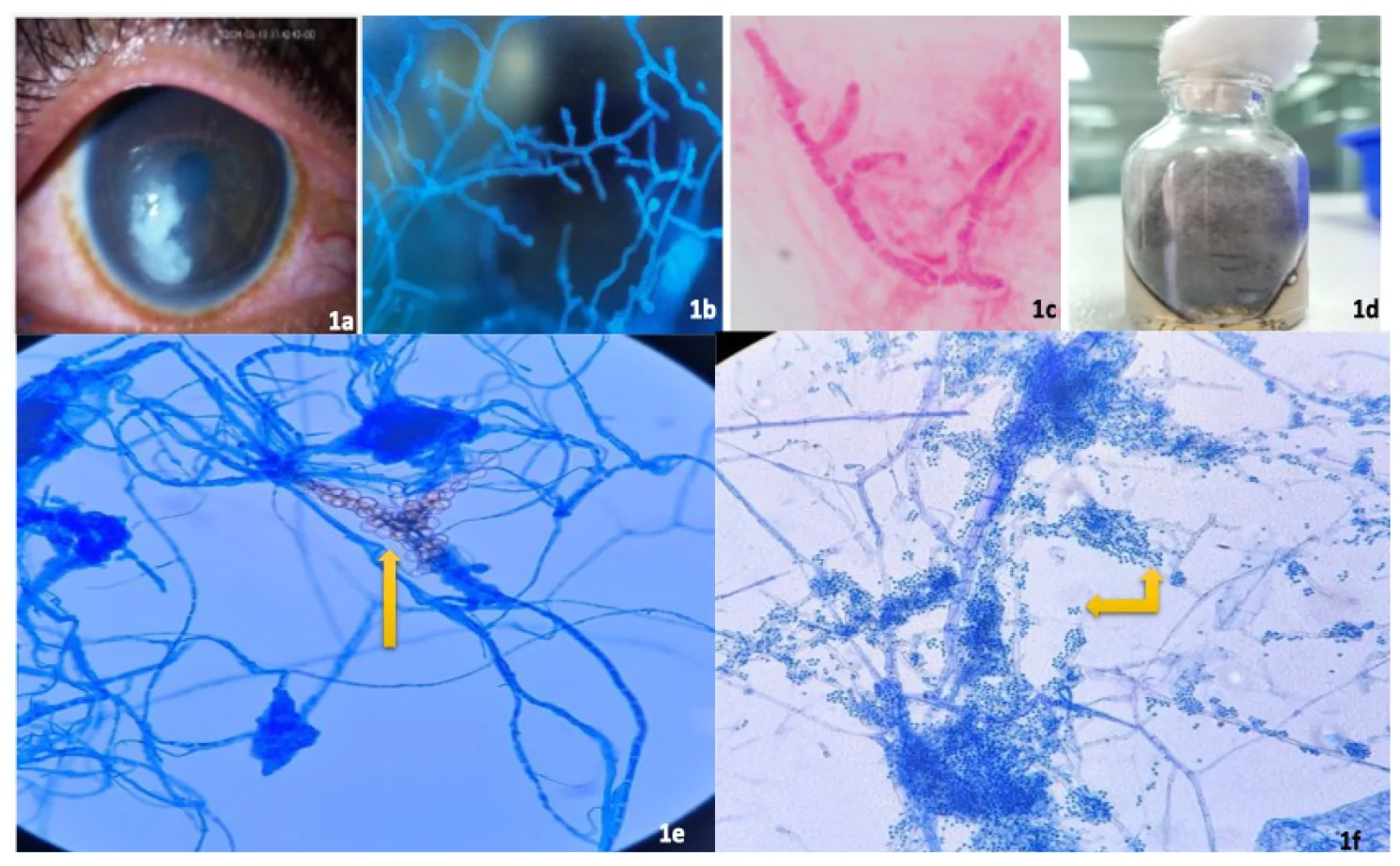
**a)** Slit lamp examination of the right eye with a 5mm x 3mm whitish superficial anterior stromal infiltrate on the cornea; **b)** Septate fungal filaments with irregular edges (KOH+CFW, 400 X); **c)** Prominent septate, branched fungal hyphae (Gram stain, 1,000 X); **d)** Rapid growth on Sabouraud dextrose agar within four days, extending to the edge of the slant by day 7; **e)** Septate fungal filaments and the presence of microsclerotia (shown by arrow) in some fields (lactophenol cotton blue, 400 X); **f)** One-celled hyaline conidia subspherical to ellipsoidal 2.5-3.5x1.5-2.5 µm adhering in slimy heads (shown by arrow) (lactophenol cotton blue, 400 X).

All media showed growth of small, feathery, appressed fungal colonies within 24 h of incubation. Within 7-10 days, colonies on SDA and PDA turned into darkly pigmented greyish black, with an olivaceous-black color on the reverse (Figure 1d). Upon microscopic examination using lactophenol cotton blue stain, the colony from SDA revealed the presence of septate filaments along with characteristic bulbils (compact clusters of thick-walled swollen fungal hyphae) in some fields (Figure 1e). One-celled hyaline conidia, subspherical to ellipsoidal, 2.5-3.5x1.5-2.5 µm, adhering in slimy heads, were also noticed (Figure 1f).

To confirm the identity of the fungus, sequencing of the internal transcribed spacer (ITS 1 and 2) region was conducted using Oxford Nanopore Technology (ONT) [7]. The culture was subjected to DNA extraction using a QiAmp DNA kit (Qiagen, Hilden, Germany, Cat. No. 51306) per the protocol provided by the manufacturer. Briefly, the fungal culture was subjected to lysis using ATL buffer and proteinase K and incubated at 56 °C for 45 min with intermittent vortex cycles. This was followed by the addition of AL reagent and incubation at 70 °C for 15 min. The lysed solution was then mixed with ice-cold molecular-grade absolute ethanol and passed through a spin column prewashed with washing solutions Wash Buffer I and Wash Buffer II, after which the DNA was eluted in 100 µL of elution buffer.

### Internal transcribed spacer region amplification by Nanopore sequencing

The ITS region from the extracted DNA was amplified using the Infexn^TM^ kit (Haystack Analytics Pvt. Ltd., Mumbai, India, Cat. No. 02ID11AMR) [7]. It was further processed by the ONT-compatible library preparation as per the manufacturer’s protocol [ONT, Oxford, UK, Cat. No. SQK-NBD 114.96]. The amplicons were processed to generate blunt ends and dA tailing using the End Repair/dA Tailing Module (New England Biolabs, Ipswich, MA, USA, Cat. No. E7546). The treated amplicons were subsequently ligated with unique barcodes (ONT, Oxford, UK, Cat. No. SQK-NBD 114.96) using the Blunt/TA Ligation Master Mix (New England Biolabs, Ipswich, MA, USA, Cat. No. M0367) [7]. These barcoded amplicons were subjected to an adapter (ONT Cat. No. EXP-NBA114) ligation using the Quick Ligation Module (New England Biolabs, Ipswich, MA, USA, Cat. No. E6056).

The library was sequenced using the ONT MinION Mk1b sequencing platform (ONT, Oxford, UK, Cat. No. Min-101B). The sequencing data were analyzed using the ΩID software (submitted in GenBank BioProject PRJNA1210108) and identified the isolate as *Podospora bulbillosa* with a mean sequence identity of 92.5% (min: 87.4% - max: 97.3%) to the reference across all reads.

### Internal transcribed spacer region and 28S gene amplification by Sanger sequencing

To confirm the correct identification, the complete ITS region and additionally the 28S gene were amplified by Sanger sequencing as described by Das et al. [8]. Briefly, after the extraction of fungal DNA using a commercial DNA extraction kit (QIAamp® DNA Mini Kit), polymerase chain reactions were performed for the complete ITS region and the large subunit 28S gene. BigDye Terminator Cycle Sequencing Kit version 3.1 (Applied Biosystems, Foster City, CA) was used for Sanger sequencing. Moreover, SeqMan Lasergene version 7.0.0 (DNAstar, Madison, WI) was used for generating the consensus sequence. Trimmed final sequences were compared with the GenBank DNA database, the ISHAM ITS database, and the CBS database. The final ITS and 28S sequences were submitted to the NCBI GenBank database (PX588566.1 and PX588565.1 respectively). The ITS sequence BLAST result showed > 98% identity with 99-100% query coverage, and the 28S sequence showed > 99% identity with 100% query coverage for both *P. bulbillosa* and *P. equi*.

### Phylogenetic analysis

The two dematiaceous fungi, *P. bulbillosa* and *Papulaspora equi*, are very closely related and can not be differentiated by macroscopic (similar colony morphology) and microscopic (both produce morphologically identical features, such as bulbils) means. Both of their ITS and 28S sequences show a high degree of similarity. Hence, phylogenetic analysis was imperative to specify the genus.

For ITS phylogeny, 100 reference sequences of the ITS gene of *P. bulbillosa* (including the ex-type strain *P. bulbillosa* 304.90), five reference sequences of ex-type Papulaspora equi strains, and 13 reference sequences of ex-type strains of other *Podospora species*, along with *Cyberlindnera jadinii* (Order: Phaffomycetales) and *Diaporthe phoenicicola* (Order: Diaporthales) as outgroups, were retrieved from GenBank.

For 28S phylogeny, additionally, 25 reference sequences (6 reference sequences of *P. bulbillosa*, including ex-type strain 304.90; 4 of *P. equi*, 15 of other *Podopsora* species) and *Aspergillus flavus* and *Curvularia hawaiiensis* as outgroups were taken from GenBank.

Sequences were aligned using ClustalX 2.1. Maximum likelihood phylogenetic trees were constructed in MEGA X version 10.2.6, using the Jukes–Cantor model with 1,000 bootstrap replicates (Figure 2a and 2b).

**Figure 2:**
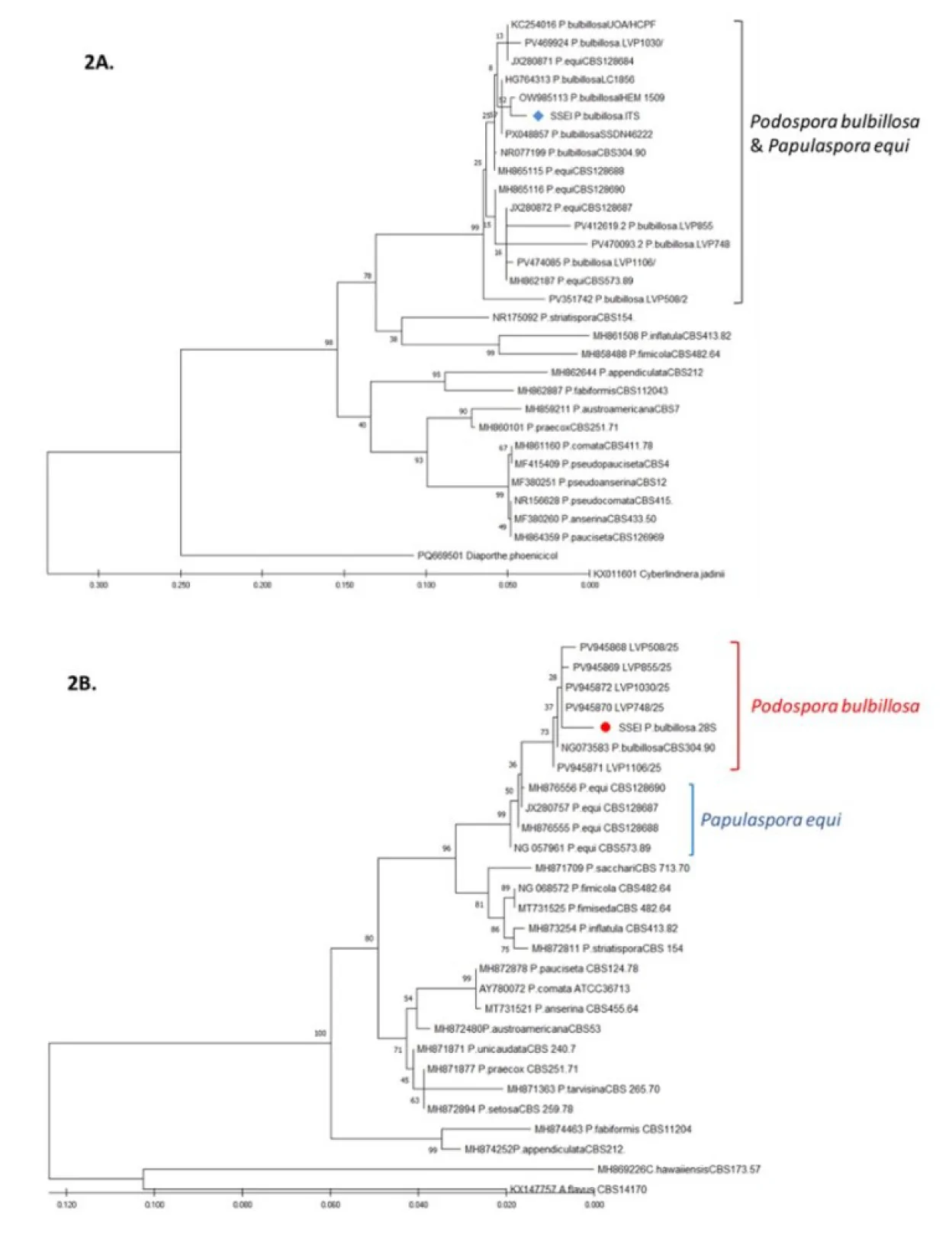
Maximum likelihood phylogenetic tree based on 28S rDNA sequences constructed in MEGA X (version 10.2.6) using the Jukes–Cantor model with 1,000 bootstrap replicates. The dataset includes 25 reference sequences (6 of *Podospora bulbillosa* including ex-type strain 304.90; 4 of *Podopsora equi*; and 15 of other *Podopsora species*) with *Aspergillus flavus* and *Curvularia*

The ITS sequence phylogeny could not differentiate between *P. bulbillosa* and *P. equi* strains, whereas both fungi were placed in two distinct but closely related clusters observed in the 28S phylogeny. In 28S phylogeny, our isolate closely matched the ex-type strain of *P. bulbillosa* 304.90 and formed a single cluster with other *P. bulbillosa* isolates reported from Southern India (Das et al. 2025), showing approximately 73% sequence identity. Hence, the isolate was identified as *P. bulbillosa*.

## Discussion

This study aimed to report a case of fungal keratitis caused by *Podospora bulbillosa* (formerly *Cladorrhinum bulbillosum*), an endophytic fungus. Podospora species are typically saprobic, found on dung and plant material in soil [6]. In 1979, the first case of *Podospora sp.* was reported from Argentina, where a 12-year-old boy, working with horses, developed fungal keratitis by foreign body trauma [9]. Later, it was isolated from the soil of an abandoned field in Dakhla Oasis, Western Desert, Egypt, and classified as *Cladorrhinum bulbillosum* by Mouchacca and Gams in 1993 [10].

This environmental strain still remains the only ex-type strain deposited in the NCBI GenBank database. After that, a total of 12 fungal keratitis cases were reported from India [8, 9, 11-13], one of them by another species of the genus *Podospora*, *P. austroamericana* [14]. The first case in India was from a 42-year-old male farmer with a history of liver cirrhosis, who had been consuming alcohol for 20 years [11]. In the present study, corneal trauma due to a foreign body was identified as the route of fungal entry.

Except for three of the earlier cases, all had a history of trauma. None of the cases had any significant association with any systemic illness except the first case from India, where the patient had compromised immunity due to liver cirrhosis [11]. In the present study, after 3 days of hourly administration of 5% topical natamycin, the ulcer showed signs of peripheral scarring and reduction in infiltrate density. The treatment was tapered over 4 weeks, and the lesion completely healed with scarring. The final best corrected visual acuity improved to 20/30 in the right eye.

The first case from Argentina was successfully treated with miconazole. All cases, except two, showed complete resolution [8,11]. In the first case from India, topical natamycin, fluconazole, and oral fluconazole failed to heal the ulcer due to compromised immunity of the patient, resulting in a poor prognosis, despite the fungal isolate being susceptible to antifungal treatment [11].

Earlier, *Podospora* was a member of the family *Lasiosphaeriaceae*; later, this Ascomycota was reclassified as a member of the family *Podosporaceae* within the order *Sordariales*. This reclassification resulted from a phylogenetic re-evaluation of the genus *Thielavia* [15]. *Podospora* sp. are primarily distinguished by their ability to produce microsclerotia and the tufted arrangement of conidiophores forming bulbils. Similar microscopic features are also observed in the fungus *Papulaspora equi*. These fungi do not produce characteristic conidia or spores, which is the distinguishing feature of *Podospora* sp. Hence, conventional microbiological techniques are not sufficient to differentiate between these two fungi.

In 28S phylogeny, although our *P. bulbillosa* isolate closely matched with *P. bulbillosa* ex-type strain 304.90, which was found in desert soil in Egypt, the bootstrap percentage similarity was comparatively lower than the acceptable range (more than 95-98%). A recent study from India by Das et al. suggested that the low degree of sequence similarity may be explained by the fact that only one ex-type strain (both in the case of ITS and 28S) has been deposited in NCBI GenBank so far. Moreover, this strain was isolated from a different geographical location. They also mentioned inaccurate sequence deposition in GenBank due to incorrect genus-level identification, particularly involving *Papulaspora equi*, may be another possible reason interfering with the lower identity score [8].

Except for one, all *P. bulbillosa* cases had been reported from India, which underscores the possible epidemiological burden of this rare yet emerging fungus in the Indian subcontinent. Such underreporting could be attributed to the lack of knowledge about this rare dematiaceous mold and its non-sporulating nature, resource-limited laboratories mostly relying on conventional identification modalities, and lack of molecular sequencing practice. Whole genome sequence-based studies on *P. bulbillosa* from different parts of India are warranted for a clear understanding about the taxonomic classification and identification of this newly emerging fungus.

## Conclusion

The case reported here emphasizes the susceptibility of the cornea to rare fungal pathogens. Recovery of the patient with natamycin highlights the importance of timely selection of appropriate antifungal therapy.
